# Chloridobis(dimethyl­glyoximato-κ^2^ *N*,*N*′)(ethyl pyridine-3-carboxyl­ate-κ*N*)cobalt(III)

**DOI:** 10.1107/S1600536811051397

**Published:** 2011-12-07

**Authors:** Ning Wang, Xuzhuo Sun, Dongjin Wan, Jing Chen, Bo Li

**Affiliations:** a Henan University of Technology, School of Chemistry and Chemical Engineering, Zhengzhou 450001, People’s Republic of China

## Abstract

In the title compound, [Co(C_4_H_7_N_2_O_2_)_2_Cl(C_8_H_9_NO_2_)], which was prepared as a model complex of vitamin B_12_, the Co^III^ atom, which is linked to four N atoms of the pseudo-macrocyclic (dmgH)_2_ ligand (dmgH is dimethyl­glyoximate) in the equatorial plane and one Cl^−^ anion and one N atom of ethyl nicotinate in apical positions, displays an approximately octa­hedral coordination. The Co atom is 0.0187 (8) Å out of the mean plane of the four equatorial N atoms. The structure has an O⋯H⋯O bridge, which is very common in cobaloxime derivatives, with O⋯H distances of 1.24 (2) and 1.25 (2) Å.

## Related literature

For background to the chemistry of cobaloximes, see: Schrayzer (1968 ▶); Zangrando *et al.* (2003 ▶). For applications of cobaloximes in proton reduction, see: Raza­vet *et al.* (2005 ▶). For related structures, see: Mandal & Gupta (2005 ▶, 2007 ▶); Bhuyan *et al.* (2007 ▶); Dutta *et al.* (2009 ▶). For NMR research on O⋯H⋯O bridges, see: Bakac & Espenson (1984 ▶). For deprotonation of O⋯H⋯O bridges by BF_3_·Et_2_O, see: Magnuson & Weber (1974 ▶). 
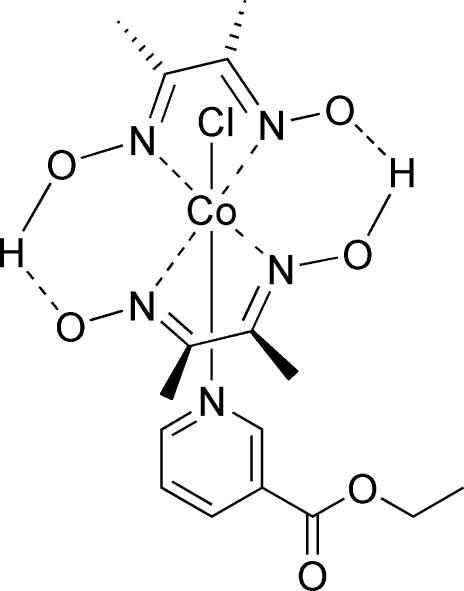


## Experimental

### 

#### Crystal data

[Co(C_4_H_7_N_2_O_2_)_2_Cl(C_8_H_9_NO_2_)]
*M* *_r_* = 475.77
Monoclinic, 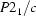

*a* = 8.1961 (11) Å
*b* = 14.2224 (19) Å
*c* = 17.365 (2) Å
β = 98.340 (2)°
*V* = 2002.8 (5) Å^3^
*Z* = 4
Mo *K*α radiation
μ = 1.03 mm^−1^
*T* = 293 K
0.32 × 0.15 × 0.06 mm

#### Data collection

Bruker APEXII area-detector diffractometer
Absorption correction: multi-scan (*SADABS*; Bruker, 2001 ▶) *T*_min_ = 0.830, *T*_max_ = 0.940
9364 measured reflections
3532 independent reflections
3104 reflections with *I* > 2σ(*I*)
*R*_int_ = 0.020

#### Refinement

*R*[*F* ^2^ > 2σ(*F* ^2^)] = 0.028
*wR*(*F* ^2^) = 0.076
*S* = 1.10
3532 reflections
272 parameters
H atoms treated by a mixture of independent and constrained refinement
Δρ_max_ = 0.27 e Å^−3^
Δρ_min_ = −0.28 e Å^−3^

### 

Data collection: *APEX2* (Bruker, 2007 ▶); cell refinement: *SAINT-Plus* (Bruker, 2007 ▶); data reduction: *SAINT-Plus*; program(s) used to solve structure: *SHELXS97* (Sheldrick, 2008 ▶); program(s) used to refine structure: *SHELXL97* (Sheldrick, 2008 ▶); molecular graphics: *SHELXTL* (Sheldrick, 2008 ▶); software used to prepare material for publication: *SHELXL97*.

## Supplementary Material

Crystal structure: contains datablock(s) I, global. DOI: 10.1107/S1600536811051397/vn2023sup1.cif

Structure factors: contains datablock(s) I. DOI: 10.1107/S1600536811051397/vn2023Isup2.hkl

Additional supplementary materials:  crystallographic information; 3D view; checkCIF report

## Figures and Tables

**Table 1:** Selected bond lengths (Å)

Co1—Cl1	2.2326 (6)
Co1—N1	1.8925 (16)
Co1—N2	1.8872 (16)
Co1—N3	1.8970 (16)
Co1—N4	1.9020 (16)
Co1—N5	1.9701 (15)

**Fig. 1.**
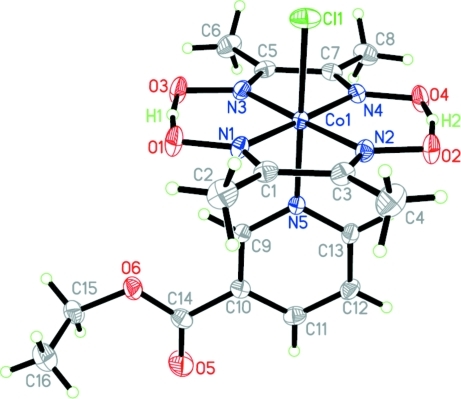
The molecular structure of the title compound. Displacement ellipsoids are drawn at the 30% probability level.

## supplementary crystallographic information

### Comment

Cobaloximes have been extensively used to mimic the vitamin B_12_ coenzyme (Schrayzer, 1968). Recently they have been also used to catalyze the proton reduction as a function model of hydrogenase (Razavet *et al.*, 2005). The cobalt atom in the title compound, [Co(dmgH)_2_(3-(COOEt)C_5_H_4_N)Cl], is in a distorted octahedral geometry by four nitrogen atoms of the pseudomacrocyclic (dmgH)_2_ ligand in the equatorial plane and by one chlorine atom and a nitrogen atom of ethyl nicotinate, respectively, in mutually *trans* positions (N5—Co—Cl1 = 179.88° (5)). The mean Co—N bond length is 1.8944 Å (16). The mean O—O distance is 2.513 Å (3). The Co atom is 0.0187 Å (8) out of the mean plane of the four nitrogen atoms. The plane of the four nitrogen atoms is practically planar. The O2···H2···O4 bridge in the structure is very common in cobaloxime derivatives (Mandal & Gupta, 2005, 2007; Bhuyan *et al.*, 2007; Dutta *et al.*, 2009). The presence of the O···H···O bridging moieties in cobaloxime derivatives ensures co-planarity of the two molecules of ligand and promotes the stability of the cobaloxime molecule (Zangrando *et al.*, 2003). The existence of O···H···O bridging is supported by NMR data and further substantiated by their chemical behavior with BF_3_.Et_2_O in readily forming an O—BF_2_—O system by deprotonation of an O···H···O bridge (Magnuson & Weber, 1974; Bakac & Espenson, 1984). The other O···H···O (O1···H1···O3) group is less bridging than  the O2···H2···O4 group since H1 is substantially close to O3 than to O1 (O3—H1 = 1.03 (3) Å)

### Experimental

Co(dmgH)(dmgH_2_)Cl, (3.6 g, 0.01 mol) and ethyl nicotinate (3.0 g, 0.02 mol) were added to chloroform (90 ml). The suspension was stirred for 20 minutes. Water (30 ml) was then added to the flask and the mixture was vigorously stirred for 2 h. The aqueous layer was discarded and the chloroform layer filtered and extracted with water until the washings were nearly colorless. The solution was reduced in volume and the product precipitated by addition of ethanol (95%); yield 69%. Brown single crystals of [Co(dmgH)_2_(3-(COOEt)C_5_H_4_N)Cl] were recrystallized from the solution CHCl_3_/acetone (v:v = 1:1). ^1^H NMR(400 MHz, CDCl_3_): δ 8.85 (s, 1 H, Ha), 8.44 (d, 5.6 Hz, 1 H, Hb), 8.30 (d, 8 Hz, 1 H, Hc), 7.26 (dd, 6 Hz, 8.4 Hz, 1 H, Hd), 4.42 (q, 7.2 Hz, 2 H, CH_2_), 2.40 (s, 12 H, N=CCH_3_), 1.42 (t, 7.2 Hz, 3 H, CH_3_).

### Refinement

H1 and H2 were located from the difference Fourier map and there positions were refined freely. Other hydrogen atoms were placed in calculated positions and refined as riding with C—H = 0.93 Å (CH) and 0.97 Å (CH_3_). The isotropic atomic displacement parameters of the the protons were constrained as follows: *U*_iso_(H) = 1.5*U*_eq_(C) for the methyl group and *U*_iso_(H) = 1.2*U*_eq_(C, O) for the others.

### Crystal data

**Table:**

[Co(C_4_H_7_N_2_O_2_)_2_Cl(C_8_H_9_NO_2_)]	*F*(000) = 984
*M**_r_* = 475.77	*D*_x_ = 1.578 Mg m^−^^3^
Monoclinic, *P*2_1_/*c*	Mo *K*α radiation, λ = 0.71073 Å
Hall symbol: -P 2ybc	Cell parameters from 4611 reflections
*a* = 8.1961 (11) Å	θ = 2.4–27.8°
*b* = 14.2224 (19) Å	µ = 1.03 mm^−^^1^
*c* = 17.365 (2) Å	*T* = 293 K
β = 98.340 (2)°	Needle, brown
*V* = 2002.8 (5) Å^3^	0.32 × 0.15 × 0.06 mm
*Z* = 4	

### Data collection

**Table:**

Bruker APEXII area-detector diffractometer	3532 independent reflections
Radiation source: fine-focus sealed tube	3104 reflections with *I* > 2σ(*I*)
graphite	*R*_int_ = 0.020
phi and ω scans	θ_max_ = 25.0°, θ_min_ = 1.9°
Absorption correction: multi-scan (*SADABS*; Bruker, 2001)	*h* = −9→9
*T*_min_ = 0.830, *T*_max_ = 0.940	*k* = −16→12
9364 measured reflections	*l* = −20→20

### Refinement

**Table:**

Refinement on *F*^2^	Primary atom site location: structure-invariant direct methods
Least-squares matrix: full	Secondary atom site location: difference Fourier map
*R*[*F*^2^ > 2σ(*F*^2^)] = 0.028	Hydrogen site location: inferred from neighbouring sites
*wR*(*F*^2^) = 0.076	H atoms treated by a mixture of independent and constrained refinement
*S* = 1.10	*w* = 1/[σ^2^(*F*_o_^2^) + (0.0374*P*)^2^ + 0.6155*P*] where *P* = (*F*_o_^2^ + 2*F*_c_^2^)/3
3532 reflections	(Δ/σ)_max_ = 0.005
272 parameters	Δρ_max_ = 0.27 e Å^−^^3^
0 restraints	Δρ_min_ = −0.28 e Å^−^^3^

### Special details

**Table:**

Geometry. All esds (except the esd in the dihedral angle between two l.s. planes) are estimated using the full covariance matrix. The cell esds are taken into account individually in the estimation of esds in distances, angles and torsion angles; correlations between esds in cell parameters are only used when they are defined by crystal symmetry. An approximate (isotropic) treatment of cell esds is used for estimating esds involving l.s. planes.
Refinement. Refinement of *F*^2^ against ALL reflections. The weighted *R*-factor *wR* and goodness of fit *S* are based on *F*^2^, conventional *R*-factors *R* are based on *F*, with *F* set to zero for negative *F*^2^. The threshold expression of *F*^2^ > σ(*F*^2^) is used only for calculating *R*-factors(gt) *etc*. and is not relevant to the choice of reflections for refinement. *R*-factors based on *F*^2^ are statistically about twice as large as those based on *F*, and *R*- factors based on ALL data will be even larger.

### Fractional atomic coordinates and isotropic or equivalent isotropic displacement parameters (Å^2^)

**Table:**

	*x*	*y*	*z*	*U*_iso_*/*U*_eq_	
Co1	0.35812 (3)	0.472077 (17)	0.212009 (14)	0.02362 (10)	
Cl1	0.21204 (7)	0.50607 (5)	0.09665 (3)	0.04574 (16)	
N3	0.2327 (2)	0.56109 (11)	0.25989 (9)	0.0296 (4)	
N1	0.5278 (2)	0.55203 (11)	0.18744 (9)	0.0291 (4)	
N4	0.1851 (2)	0.39228 (11)	0.23476 (9)	0.0288 (4)	
O1	0.53203 (19)	0.64264 (10)	0.20602 (9)	0.0436 (4)	
N5	0.48695 (18)	0.44181 (11)	0.31377 (9)	0.0240 (3)	
C1	0.6351 (2)	0.51383 (15)	0.14900 (11)	0.0316 (5)	
N2	0.4780 (2)	0.38275 (11)	0.16259 (9)	0.0296 (4)	
O3	0.2744 (2)	0.65352 (10)	0.26840 (10)	0.0438 (4)	
H1	0.381 (3)	0.6557 (17)	0.2433 (14)	0.053*	
O4	0.17824 (19)	0.30074 (10)	0.21648 (9)	0.0418 (4)	
O2	0.4320 (2)	0.29203 (10)	0.15452 (9)	0.0431 (4)	
H2	0.298 (3)	0.2881 (17)	0.1809 (14)	0.052*	
C7	0.0736 (2)	0.43004 (15)	0.27068 (11)	0.0300 (4)	
C5	0.1010 (2)	0.53125 (14)	0.28465 (11)	0.0302 (5)	
C3	0.6056 (2)	0.41350 (15)	0.13459 (11)	0.0332 (5)	
C10	0.6455 (2)	0.48779 (14)	0.43410 (11)	0.0298 (4)	
C13	0.5055 (2)	0.35154 (13)	0.33651 (12)	0.0304 (4)	
H13A	0.4576	0.3047	0.3032	0.036*	
C2	0.7698 (3)	0.56681 (18)	0.11945 (14)	0.0479 (6)	
H2A	0.7457	0.5722	0.0639	0.072*	
H2B	0.8722	0.5340	0.1332	0.072*	
H2C	0.7783	0.6284	0.1422	0.072*	
C9	0.5572 (2)	0.50894 (13)	0.36194 (11)	0.0264 (4)	
H9A	0.5463	0.5715	0.3464	0.032*	
O6	0.6991 (2)	0.64724 (10)	0.45873 (8)	0.0423 (4)	
O5	0.7992 (3)	0.54489 (13)	0.55052 (10)	0.0663 (6)	
C11	0.6623 (3)	0.39457 (15)	0.45707 (12)	0.0365 (5)	
H11A	0.7198	0.3789	0.5055	0.044*	
C14	0.7245 (3)	0.56221 (16)	0.48794 (12)	0.0360 (5)	
C12	0.5929 (3)	0.32574 (15)	0.40745 (12)	0.0366 (5)	
H12A	0.6045	0.2627	0.4213	0.044*	
C6	−0.0121 (3)	0.59176 (18)	0.32237 (14)	0.0481 (6)	
H6A	−0.0050	0.5750	0.3763	0.072*	
H6B	−0.1231	0.5830	0.2970	0.072*	
H6C	0.0189	0.6565	0.3181	0.072*	
C8	−0.0657 (3)	0.37698 (18)	0.29583 (14)	0.0450 (6)	
H8A	−0.0690	0.3147	0.2743	0.068*	
H8B	−0.1674	0.4088	0.2778	0.068*	
H8C	−0.0506	0.3732	0.3516	0.068*	
C15	0.7661 (3)	0.72628 (16)	0.50703 (13)	0.0447 (6)	
H15A	0.7672	0.7105	0.5615	0.054*	
H15B	0.6954	0.7807	0.4954	0.054*	
C4	0.7100 (3)	0.35388 (19)	0.09048 (15)	0.0545 (7)	
H4A	0.6836	0.2888	0.0968	0.082*	
H4B	0.8242	0.3643	0.1100	0.082*	
H4C	0.6893	0.3702	0.0363	0.082*	
C16	0.9359 (3)	0.74998 (19)	0.49341 (15)	0.0545 (7)	
H16A	0.9764	0.8019	0.5260	0.082*	
H16B	0.9347	0.7668	0.4398	0.082*	
H16C	1.0064	0.6965	0.5057	0.082*	

### Atomic displacement parameters (Å^2^)

**Table:**

	*U*^11^	*U*^22^	*U*^33^	*U*^12^	*U*^13^	*U*^23^
Co1	0.02584 (16)	0.02073 (15)	0.02457 (15)	0.00085 (10)	0.00463 (11)	−0.00105 (10)
Cl1	0.0416 (3)	0.0654 (4)	0.0287 (3)	0.0097 (3)	−0.0002 (2)	0.0059 (3)
N3	0.0331 (9)	0.0236 (9)	0.0318 (9)	0.0045 (7)	0.0038 (7)	0.0002 (7)
N1	0.0338 (9)	0.0213 (8)	0.0328 (9)	0.0000 (7)	0.0068 (7)	0.0037 (7)
N4	0.0295 (9)	0.0256 (9)	0.0302 (9)	−0.0030 (7)	0.0007 (7)	−0.0022 (7)
O1	0.0527 (10)	0.0208 (8)	0.0608 (10)	−0.0049 (7)	0.0199 (8)	0.0019 (7)
N5	0.0252 (8)	0.0215 (8)	0.0260 (8)	0.0002 (6)	0.0064 (6)	−0.0001 (6)
C1	0.0310 (11)	0.0358 (12)	0.0284 (10)	0.0015 (9)	0.0057 (9)	0.0075 (9)
N2	0.0364 (10)	0.0243 (9)	0.0286 (8)	0.0022 (7)	0.0059 (7)	−0.0035 (7)
O3	0.0500 (10)	0.0216 (7)	0.0626 (10)	0.0031 (7)	0.0173 (8)	−0.0051 (7)
O4	0.0425 (9)	0.0280 (8)	0.0559 (10)	−0.0114 (7)	0.0100 (8)	−0.0108 (7)
O2	0.0538 (10)	0.0254 (8)	0.0521 (9)	−0.0034 (7)	0.0148 (8)	−0.0141 (7)
C7	0.0255 (10)	0.0378 (12)	0.0261 (10)	−0.0003 (9)	0.0016 (8)	0.0005 (8)
C5	0.0286 (10)	0.0358 (12)	0.0260 (10)	0.0082 (8)	0.0035 (8)	0.0020 (8)
C3	0.0347 (11)	0.0372 (12)	0.0289 (10)	0.0070 (9)	0.0088 (9)	0.0004 (9)
C10	0.0282 (10)	0.0330 (11)	0.0285 (10)	0.0002 (8)	0.0047 (8)	−0.0006 (8)
C13	0.0333 (11)	0.0223 (10)	0.0359 (11)	0.0002 (8)	0.0063 (9)	0.0005 (8)
C2	0.0439 (13)	0.0537 (15)	0.0499 (14)	−0.0018 (11)	0.0197 (11)	0.0134 (12)
C9	0.0271 (10)	0.0242 (10)	0.0284 (10)	0.0008 (8)	0.0058 (8)	0.0005 (8)
O6	0.0549 (10)	0.0320 (8)	0.0364 (8)	−0.0040 (7)	−0.0056 (7)	−0.0065 (7)
O5	0.0923 (15)	0.0546 (11)	0.0409 (10)	−0.0077 (10)	−0.0283 (10)	0.0029 (8)
C11	0.0361 (12)	0.0410 (13)	0.0313 (11)	0.0038 (9)	0.0012 (9)	0.0082 (9)
C14	0.0354 (12)	0.0421 (13)	0.0294 (11)	−0.0024 (10)	0.0014 (9)	−0.0033 (9)
C12	0.0402 (12)	0.0271 (11)	0.0420 (12)	0.0036 (9)	0.0043 (10)	0.0091 (9)
C6	0.0463 (14)	0.0515 (15)	0.0493 (14)	0.0159 (11)	0.0158 (11)	−0.0007 (11)
C8	0.0324 (12)	0.0562 (15)	0.0472 (13)	−0.0083 (11)	0.0084 (10)	0.0013 (11)
C15	0.0485 (14)	0.0396 (13)	0.0434 (13)	−0.0036 (11)	−0.0016 (11)	−0.0170 (10)
C4	0.0595 (16)	0.0564 (16)	0.0530 (15)	0.0150 (13)	0.0271 (13)	−0.0061 (12)
C16	0.0541 (15)	0.0545 (16)	0.0559 (15)	−0.0110 (12)	0.0109 (12)	−0.0137 (13)

### Geometric parameters (Å, °)

**Table:**

Co1—Cl1	2.2326 (6)	C13—C12	1.382 (3)
Co1—N1	1.8925 (16)	C13—H13A	0.9300
Co1—N2	1.8872 (16)	C2—H2A	0.9600
Co1—N3	1.8970 (16)	C2—H2B	0.9600
Co1—N4	1.9020 (16)	C2—H2C	0.9600
Co1—N5	1.9701 (15)	C9—H9A	0.9300
N3—C5	1.291 (3)	O6—C14	1.316 (3)
N3—O3	1.361 (2)	O6—C15	1.461 (2)
N1—C1	1.298 (3)	O5—C14	1.193 (3)
N1—O1	1.328 (2)	C11—C12	1.372 (3)
N4—C7	1.296 (3)	C11—H11A	0.9300
N4—O4	1.339 (2)	C12—H12A	0.9300
N5—C9	1.343 (2)	C6—H6A	0.9600
N5—C13	1.345 (2)	C6—H6B	0.9600
C1—C3	1.463 (3)	C6—H6C	0.9600
C1—C2	1.488 (3)	C8—H8A	0.9600
N2—C3	1.292 (3)	C8—H8B	0.9600
N2—O2	1.346 (2)	C8—H8C	0.9600
O3—H1	1.03 (3)	C15—C16	1.484 (3)
O4—H2	1.24 (2)	C15—H15A	0.9700
O2—H2	1.25 (2)	C15—H15B	0.9700
C7—C5	1.472 (3)	C4—H4A	0.9600
C7—C8	1.486 (3)	C4—H4B	0.9600
C5—C6	1.485 (3)	C4—H4C	0.9600
C3—C4	1.493 (3)	C16—H16A	0.9600
C10—C9	1.386 (3)	C16—H16B	0.9600
C10—C11	1.386 (3)	C16—H16C	0.9600
C10—C14	1.496 (3)		
			
N2—Co1—N1	81.59 (7)	C12—C13—H13A	118.8
N2—Co1—N3	178.54 (7)	C1—C2—H2A	109.5
N1—Co1—N3	99.23 (7)	C1—C2—H2B	109.5
N2—Co1—N4	98.39 (7)	H2A—C2—H2B	109.5
N1—Co1—N4	178.87 (7)	C1—C2—H2C	109.5
N3—Co1—N4	80.76 (7)	H2A—C2—H2C	109.5
N2—Co1—N5	90.80 (7)	H2B—C2—H2C	109.5
N1—Co1—N5	91.01 (7)	N5—C9—C10	121.97 (18)
N3—Co1—N5	90.38 (7)	N5—C9—H9A	119.0
N4—Co1—N5	90.12 (6)	C10—C9—H9A	119.0
N2—Co1—Cl1	89.12 (5)	C14—O6—C15	117.41 (17)
N1—Co1—Cl1	89.06 (5)	C12—C11—C10	119.16 (19)
N3—Co1—Cl1	89.69 (5)	C12—C11—H11A	120.4
N4—Co1—Cl1	89.81 (5)	C10—C11—H11A	120.4
N5—Co1—Cl1	179.89 (5)	O5—C14—O6	124.9 (2)
C5—N3—O3	119.37 (16)	O5—C14—C10	122.8 (2)
C5—N3—Co1	117.34 (14)	O6—C14—C10	112.29 (17)
O3—N3—Co1	123.28 (13)	C11—C12—C13	118.99 (19)
C1—N1—O1	122.39 (17)	C11—C12—H12A	120.5
C1—N1—Co1	116.07 (13)	C13—C12—H12A	120.5
O1—N1—Co1	121.49 (12)	C5—C6—H6A	109.5
C7—N4—O4	120.58 (17)	C5—C6—H6B	109.5
C7—N4—Co1	116.87 (14)	H6A—C6—H6B	109.5
O4—N4—Co1	122.54 (12)	C5—C6—H6C	109.5
N1—O1—H1	103.4 (10)	H6A—C6—H6C	109.5
C9—N5—C13	118.40 (17)	H6B—C6—H6C	109.5
C9—N5—Co1	121.94 (13)	C7—C8—H8A	109.5
C13—N5—Co1	119.66 (13)	C7—C8—H8B	109.5
N1—C1—C3	112.86 (17)	H8A—C8—H8B	109.5
N1—C1—C2	123.9 (2)	C7—C8—H8C	109.5
C3—C1—C2	123.21 (19)	H8A—C8—H8C	109.5
C3—N2—O2	121.02 (16)	H8B—C8—H8C	109.5
C3—N2—Co1	116.53 (14)	O6—C15—C16	111.49 (19)
O2—N2—Co1	122.42 (12)	O6—C15—H15A	109.3
N3—O3—H1	101.4 (14)	C16—C15—H15A	109.3
N4—O4—H2	104.4 (11)	O6—C15—H15B	109.3
N2—O2—H2	104.6 (11)	C16—C15—H15B	109.3
N4—C7—C5	112.56 (17)	H15A—C15—H15B	108.0
N4—C7—C8	123.8 (2)	C3—C4—H4A	109.5
C5—C7—C8	123.62 (18)	C3—C4—H4B	109.5
N3—C5—C7	112.44 (16)	H4A—C4—H4B	109.5
N3—C5—C6	124.2 (2)	C3—C4—H4C	109.5
C7—C5—C6	123.35 (19)	H4A—C4—H4C	109.5
N2—C3—C1	112.88 (17)	H4B—C4—H4C	109.5
N2—C3—C4	123.8 (2)	C15—C16—H16A	109.5
C1—C3—C4	123.29 (19)	C15—C16—H16B	109.5
C9—C10—C11	119.00 (19)	H16A—C16—H16B	109.5
C9—C10—C14	122.23 (19)	C15—C16—H16C	109.5
C11—C10—C14	118.77 (19)	H16A—C16—H16C	109.5
N5—C13—C12	122.48 (19)	H16B—C16—H16C	109.5
N5—C13—H13A	118.8		
